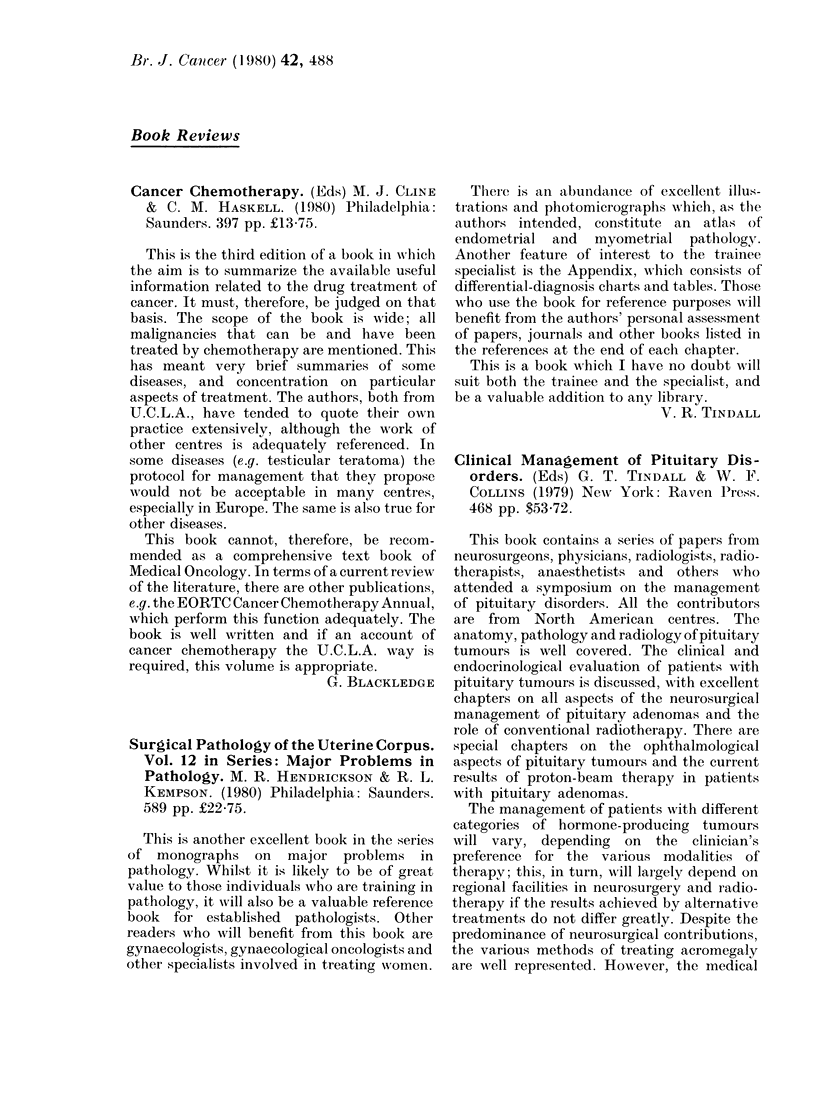# Cancer Chemotherapy

**Published:** 1980-09

**Authors:** G. Blackledge


*Br. J. Cancer* (1980) **42**, 488

### *Book Reviews*

**Cancer Chemotherapy.** (Eds) M. J. Cline & C. M. Haskell. (1980) Philadelphia: Saunders. 397 pp. £13·75.

This is the third edition of a book in which the aim is to summarize the available useful information related to the drug treatment of cancer. It must, therefore, be judged on that basis. The scope of the book is wide; all malignancies that can be and have been treated by chemotherapy are mentioned. This has meant very brief summaries of some diseases, and concentration on particular aspects of treatment. The authors, both from U.C.L.A., have tended to quote their own practice extensively, although the work of other centres is adequately referenced. In some diseases (*e.g.* testicular teratoma) the protocol for management that they propose would not be acceptable in many centres, especially in Europe. The same is also true for other diseases.

This book cannot, therefore, be recommended as a comprehensive text book of Medical Oncology. In terms of a current review of the literature, there are other publications, *e.g.* the EORTC Cancer Chemotherapy Annual, which perform this function adequately. The book is well written and if an account of cancer chemotherapy the U.C.L.A. way is required, this volume is appropriate.

G. Blackledge